# Serological response to lumpy skin disease in recovered and clinically healthy vaccinated and unvaccinated cattle of Bangladesh

**DOI:** 10.3389/fvets.2025.1535600

**Published:** 2025-02-17

**Authors:** Rokshana Parvin, Sirat Al Mim, Md. Nurul Haque, Israt Jerin, Mohammed Nooruzzaman, Md. Riabbel Hossain, Emdadul Haque Chowdhury, Anja Globig, Sascha Knauf, Eeva Tuppurainen

**Affiliations:** ^1^Department of Pathology, Faculty of Veterinary Science, Bangladesh Agricultural University, Mymensingh, Bangladesh; ^2^Institute of International Animal Health/One Health, Friedrich-Loeffler-Institute, Greifswald-Insel Riems, Germany; ^3^Faculty of Veterinary Medicine, Justus Liebig University, Giessen, Germany

**Keywords:** seromonitoring, ELISA, LSD, recovered and affected cattle, vaccinated and unvaccinated cattle

## Abstract

Lumpy skin disease (LSD) is one of the most economically important transboundary animal diseases that emerged in Bangladesh in 2019. It has a significant economic impact on household cattle owners in rural settings in Bangladesh. A cross-sectional study was undertaken in selected areas of the Mymensingh districts of Bangladesh between July 2021 and May 2023. A total of 1,161 blood samples were collected from 105 households and four herds comprising 904 and 257 cattle, respectively. The presence of LSD virus (LSDV) antibodies in serum was detected using enzyme-linked immunosorbent assay (ELISA). The overall seroprevalence of LSD in the study area during the sampling period was 26.2% (*n* = 304/1,161; 95% confidence interval: 4.90–10.20). Based on the disease status, the seroprevalence of the recovered animal was 40.07%, significantly higher than that of unvaccinated animals that had been in contact with affected cattle but never showed any visible clinical signs of LSD (23.27%), and the seroprevalence in cattle that were showing clinical signs when serum samples were collected (18.0%). Nonetheless, seroconversion in the vaccinated population lasted 6–12 months after vaccination, and animals that recovered natural infection also exhibited measurable seroconversion up to 6 months after exposure. The study demonstrated the seroprevalence of LSD in cattle kept in rural Bangladeshi households and the duration of antibody responses in animals recovered from natural LSD infection, cattle that were clinically healthy but had circulating LSDV in the herd, and animals vaccinated with vaccines containing goat pox virus or attenuated LSDV. The results of this study help in defining an effective and feasible vaccination strategy considering the duration of immunity after vaccination or natural LSD infection.

## 1 Introduction

Lumpy skin disease (LSD) affects cattle, water buffalo, and wild ruminants such as giraffes, impalas, wildebeest, springboks, and oryxes (1) of all ages and breeds. It is an emerging, highly contagious, transboundary viral disease clinically distinguished by high fever, lymphadenopathy, nodular skin lesions, and edema in the brisket region or legs (2, 3). The disease is caused by the Lumpy Skin Disease Virus (LSDV), a double-stranded DNA Capripoxvirus belonging to the Poxviridae family (4). The disease was first discovered in 1929 in Northern Rhodesia, Zambia (5). Until 1990, the disease was only found in sub-Saharan Africa before spreading to Egypt and, eventually, the Middle East (6, 7). In the Balkan region, LSDV caused widespread outbreaks between 2015 and 2016 but due to a coordinated regional control and eradication policy, the spread of the disease was halted by the end of 2017 (8). Outbreaks were also reported in Turkey and the Russian Federation, among other places (9–12). Since then, the disease has continued its spread in Asia affecting countries such as Afghanistan, Bangladesh, Bhutan, China, India, Indonesia, Nepal, and Pakistan (13–16).

Livestock plays an essential role in agricultural production in Bangladesh because it provides the primary source of protein for human consumption, organic manure for crops, and means of transport in both rural and urban areas. According to the Economic Report of the Department of Livestock Services (DLS) of Bangladesh, 2021–2022, there are approximately 26.2 million native breeds of cattle and buffalo in the country. The contribution of livestock to the Gross Domestic Product (GDP) is around 1.90%, while the GDP growth rate of livestock is 3.10% (17). Farmers generate income through the sale of live animals and animal products. Livestock also plays a major role in the national economy as it is a major source of foreign exchange profits through the export of hides and skins. However, currently, the main obstacles to livestock production in Bangladesh include a lack of feed, animal diseases, the limited genetic potential of native livestock, and a lack of marketing infrastructure. LSD is one of the many livestock illnesses that are known to cause significant financial losses and low output in livestock in affected countries (15, 18). Due to the reduction in milk and meat production, as well as the lower fertility rates and death or veterinary treatment of severely infected animals, LSD has significant economic repercussions for the livestock sector involved in cattle rearing either by small-scale farmers or in a more intensive farm setting. The costs of obligatory control, prevention, and eradication measures, immunizations, and the effect of outbreaks on trade and mobility of live cattle contribute to economic losses. The lower commercial value or complete rejection of the permanently scarred or damaged skins from LSD-infected animals have a negative impact on the income of countries exporting cattle skins and hides (15, 18–20). Affected farmers may lose access to health care and nutritional resources due to a lack of income. Moreover, antibiotics are often used to counteract secondary infections, increasing the risk for AMR (21) Numerous techniques have been employed for LSDV diagnosis including molecular detection, ELISA, a virus neutralization test, an immune-peroxidase monolayer assay (22), an indirect fluorescent antibody test, and a skin hypersensitivity test (4, 23).

In Bangladesh, the first official outbreak of LSD was reported in August 2019 and was confined to a single district Chattogram located in the Southeastern part of the country (24). Later more outbreaks were reported across the nation (15, 18–20). Due to its widespread distribution and enormous cow population in backyard settings, the disease contributes to rising poverty and dwindling food security. Today, LSD is one of Bangladesh’s most economically significant livestock diseases (20).

Vaccination, livestock movement restrictions, quarantine, vector control, the slaughter of infected and exposed animals, as well as cleaning and disinfection of premises, are recommended for LSD control and prevention (10, 25). So far, none of those measures have been employed effectively in Bangladesh to control the disease. A locally produced attenuated goat pox virus-based vaccine and a commercially available Lumpyvax™ vaccine (MSD Animal Health, South Africa), containing LSD Neethling type strain (SIS) are currently used to vaccinate against LSDV in Bangladesh. However, vaccination coverage is minimal and the vaccines are mostly supplied on demand by farmers. The antibody response of the vaccinated herds has been rarely monitored in Bangladesh. The serological assay recommended in the World Organization of Animal Health (WOAH) Terrestrial Manual for monitoring immunity following vaccination is a virus neutralization test (VNT). The first validated enzyme-linked immunosorbent assay (ELISA) (ID.Vet) however, is commercially available for large-scale LSD surveillance (26). This ELISA can detect antibodies against capripoxviruses (LSDV, SPV, and GPV) from about 20 days to 7 months after vaccination (25, 26) with a sensitivity of 83% and specificity of 99.7% (27). Yet, the serological assay has some drawbacks, such as time-dependent outcomes (28), limited sensitivity and specificity (29), false positives and negatives, variable antibody responses, and the inability to discriminate between ongoing wild-type infections (30).

Several studies have been conducted in Bangladesh to determine the overall prevalence of LSD based on clinical observation, and the disease prevalence has varied from 13.20 to 31.50% from various locations throughout the country since 2019 (24, 31–34). Despite disease prevalence in the affected area, a significant number of cattle in the same area, including cattle from the same infected household, appear clinically healthy. These animals must be either uninfected or they are infected without showing any clinical signs (silent infection). Because cattle with silent infection may spread the virus (35), it is important to know the percentage of seroconverting animals not showing clinical signs in affected, unvaccinated herds. In addition, in this study antibody response against the LSDV is investigated, as well as the seroconversion in animals vaccinated with homologous LSDV and heterologous goat pox virus-based vaccines.

This inquiry into the seroprevalence at the individual household and at the herd level cattle (vaccinated and unvaccinated) will help to ascertain the current state of LSD in that particular area which might improve disease prevention strategies and deepen the nation’s limited understanding of LSD’s epidemiology. Our study, therefore, aimed to investigate the seroprevalence of LSD in cattle at the household and herd levels in the central Northern part of Bangladesh (Supplementary Figure S1) bordering India and to compare the antibody response between affected, recovered clinically healthy vaccinated and unvaccinated animals.

## 2 Materials and methods

### 2.1 Study area, housing, and population size

The study was conducted between July 2021 and May 2023 in different Upazilas (sub-districts) of the Mymensingh division in Bangladesh. Cattle in this study area were mostly indigenous Zebu cattle (*Bos taurus*) with a few crossbreds (a cross between Holstein Friesian and local).

In a backyard farming system, animals were kept in a loose housing system in the backyard of the owner’s house. Each household in this survey had 2–15 bovines, later referred to as “household (backyard cattle).” The selection of small-sized households was based on the number of cattle usually available in rural areas and should have a history of at least one LSD-affected animal. These animals are mostly fed on grass from nearby fields. They also get leftover food from the owner’s family, including rice, vegetables, and fruits, with a regular addition of bran. The backyard animals had no reported contact with cattle of neighboring farms or with wild ruminants.

In addition, four more advanced cattle farms located in the Mymensingh division were selected for the study. Each herd contained at least 20 cattle, housed in semi-standard and conventional barn systems, referred to here as the “herd cattle.” the selection of the small-sized herds is also based on the outbreak history and willingness to interact with the research project. The feedlot animals were given proper cattle feed and were housed following appropriate husbandry and good management practices compared to the backyard cattle. A total of 105 backyards with 904 cattle and four herds comprising 257 cattle (*n* = 1,161) were investigated. The outbreak investigations were carried out by collecting primary data from farm supervising veterinarians and farmers using a validated structured questionnaire. The questions focused on demographic information and clinical observations. The backyard cattle had no vaccination history against the diseases whereas the feedlot cattle were vaccinated with either Goatpox or Lumpyvax™ vaccine.

### 2.2 Study design and sampling strategy

A cross-sectional study was designed to collect the samples, and the individual animal in each household was considered as the sampling unit. Apart from regular sampling based on the outbreak history, this study also included multistage (repeated) sampling. Briefly, the day of the first sample collection is referred to as D0. The second samples were collected 40 days later (D40), and the last samples on day 70 (D70) from the first investigation. The reduced number in repeated sampling is attributed to the farmer’s unwillingness (they protect their afflicted animal from bleeding.) and unavailability (Farmers often sell their sick animals) of the respective specific animals. Additionally, samples were collected from vaccinated (either goat pox or LSD) animal and recovered animal (LSD natural infection). The study population was divided into the following three categories:

- Infected animals showing typical clinical signs at the time of investigation.
- Recovered animals with a history of LSDV infection within the last 12 months.
- Animals that remained clinically healthy after an outbreak in the herd or household and contact with infected animals.

**Figure 1:**
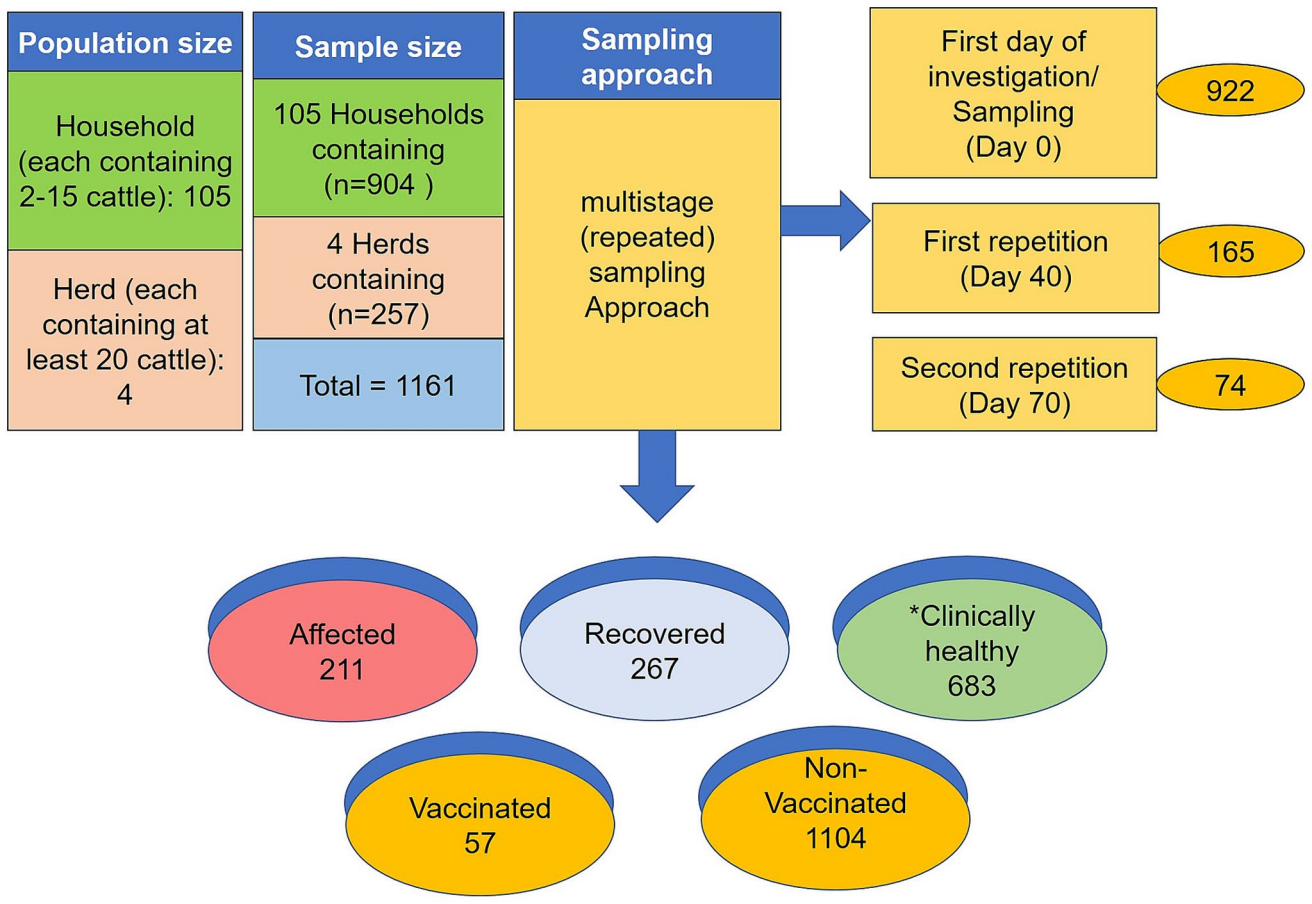
Graphic sketch of sampling size and sampling model. *Denotes the clinically healthy but in contact with affected animals. The numerical is the total number of samples collected from different stages of sampling.

The sampling size and sampling models are depicted in Figure 1. A standard questionnaire was used to collect demographic data such as age, sex, disease status, vaccination history, farming type, and contacts with other cattle of the same household or within the community. During sample collection, cattle owners were consulted to help estimate the age of each sampled animal. All the sampled cattle were grouped into three age-based categories; calf: ≤1 year; young: >1 to ≤2.5 years and adult >2.5 years (36) The selection of study areas and animals was based on the suspected cases reported by the local veterinarians and physical visits to the farms. A case was considered positive for LSD when at least one animal in the household showed febrile disease with nodular skin lesions. Samples from clinically healthy, affected, and recovered animals were collected from the household and herd cattle following a simple randomization technique.

### 2.3 Sample collections and serum preparation

In this study, a total of 1,161 blood samples were collected from 105 households and four herds. During the study period, 211 samples were collected from ongoing LSD outbreaks (affected cattle), 267 samples were recovered, and 683 samples were from clinically healthy animals that shared the same household or herd with a history of previous LSD outbreaks. Among the total samples, 922 were collected on Day 0, 165 repeated on Day 40, and 74 repeated on Day 70. For serum, approximately 8–10 mL blood was drawn from the jugular vein using disposable needles, collected in a 10 mL vacutainer tube without EDTA (BD vacutainer), and then allowed to clot at room temperature for at least 30 min on the sampling spot. Later the vacutainer was kept overnight at 4°C to allow blood to clot at a slanted position. The serum was separated, clarified by centrifugation at 3,000 for 10 min, and stored at −20°C till the tests were performed. Besides the serum samples, 210 additional samples were collected from clinically LSD-affected cattle, including 40 skin samples (scabs or nodules), 52 whole-blood samples (blood in EDTA), 40 saliva, 20 nasal swabs, 18 fecal samples, and 20 milk samples were collected from ongoing 211 LSD outbreaks at the time of sampling. A total of 100 blood samples were also collected randomly from apparently healthy cattle that were co-housed with LSD-affected cattle. The sample collection procedure was carried out following the previous protocol (19).

### 2.4 ELISA

The recommended ID Screen® Capripox double antigen multi-species (ID.Vet, Grabels, France) ELISA was used to detect antibodies against LSDV according to the manufacturer’s instructions. Briefly, the optical density was measured at 450 nm using an ELISA microplate. For each sample, the percentage OD of sample/OD of positive control (S/P percentage) was calculated using the formula suggested in the manual: S/P% = (OD sample − ODNC/ODPC − ODNC) * 100. Where OD sample is the optical density of the sample, ODPC is the optical density of positive control, and ODNC is the optical density of negative control. Samples presenting an S/P% less than 30% were considered negative while those with an S/P% greater than or equal to 30% were considered positive. Positive and negative control was incorporated in the mentioned commercial kit.

### 2.5 Molecular detection of the virus in clinically affected cattle

Total DNA was extracted from skin lesions (scabs or nodules), blood, saliva, nasal swabs, feces, and milk samples using the DNeasy Blood & Tissue Kit (Qiagen, Hilden, Germany). DNA was then tested by a Capripox generic real-time PCR (qPCR) that amplifies a part of the P32 envelope protein-gene using a previously described primer and probe mix (37, 38). The reaction mixture and thermal profile were used as described previously by Parvin (19). Briefly, the qPCR reaction was prepared in a total volume of 12.5 μL reaction consisting of 6 μL of Luna® Universal Probe qPCR Master Mix (NEB, United Kingdom), 2 μL of the primer-probe mix, 2 μL of nuclease-free water and 2.5 μL of template DNA. The PCR was carried out on QuantStudio™ 5 Real-Time PCR System (Applied Biosystems, USA). Positive detection was determined at 35 cycle threshold (Ct) values and after a clear sigmoid curve at qPCR.

### 2.6 Data analysis

Before statistical analysis, data from the farmers’ questionnaire and the laboratory results were entered, coded, and filtered in Microsoft Excel. Using a statistical analysis program in social science (SPSS), a statistical study was carried out (IBM Ver 24, USA). To assess the relationship between various factors and the frequency of LSD, logistic regression was used. The chi-square test and the variable stepwise forward multivariable logistic regression model were used to identify the most important variables. Using descriptive statistics, rates, diagrams, and charts were determined. Graphs were prepared using GraphPad Prism 9.0. One-way ANOVA with Bonferroni multiple comparison test was performed to compare the LSD antibody responses in cattle.

## 3 Results

### 3.1 Outbreak investigation

Several LSD outbreaks were reported by farmers during the summer of 2021 to the Upazila Livestock Office and Veterinary Hospitals of the Mymensingh division of Bangladesh. A cross-sectional study was designed to investigate the outbreak using a standard questionnaire. At the time of the investigation, 18.17% of animals included in this study showed ongoing clinical disease (*n* = 211), 22.99% had recovered from recent infection (*n* = 267) and 58.82% of in-contact animals remained clinically healthy (*n* = 683). Among the clinically affected and recovered animals (*n* = 478), 65.2% were female and 34.7% were male cattle. Of these, cows (39.4%) and calves (30%) were mostly affected compared to heifers (16.8%) and bulls (13.1%). Clinical signs included nodular skin lesions on the body, fever, lameness, and swelling in the brisket region, joints, and lymph nodes in addition, respiratory distress was particularly detected in calves. Some affected cattle had received preventive, and symptomatic treatment with painkillers, antipyretics, and antihistamines at the initial stage, if the lump ruptured then antibiotics, and local antiseptic spry were usually suggested, while some remained untreated. The disease period usually varied from 15 days to as many as 52 days (Mean 21.35 and SEM 0.46). Our previous study describes the clinical disease from the same study area in detail (19). To understand the disease pattern and antibody responses in clinically healthy animals the study further focused on seroprevalence analysis at different parameters.

### 3.2 Seroprevalence of LSD affected area and population

**Figure 2:**
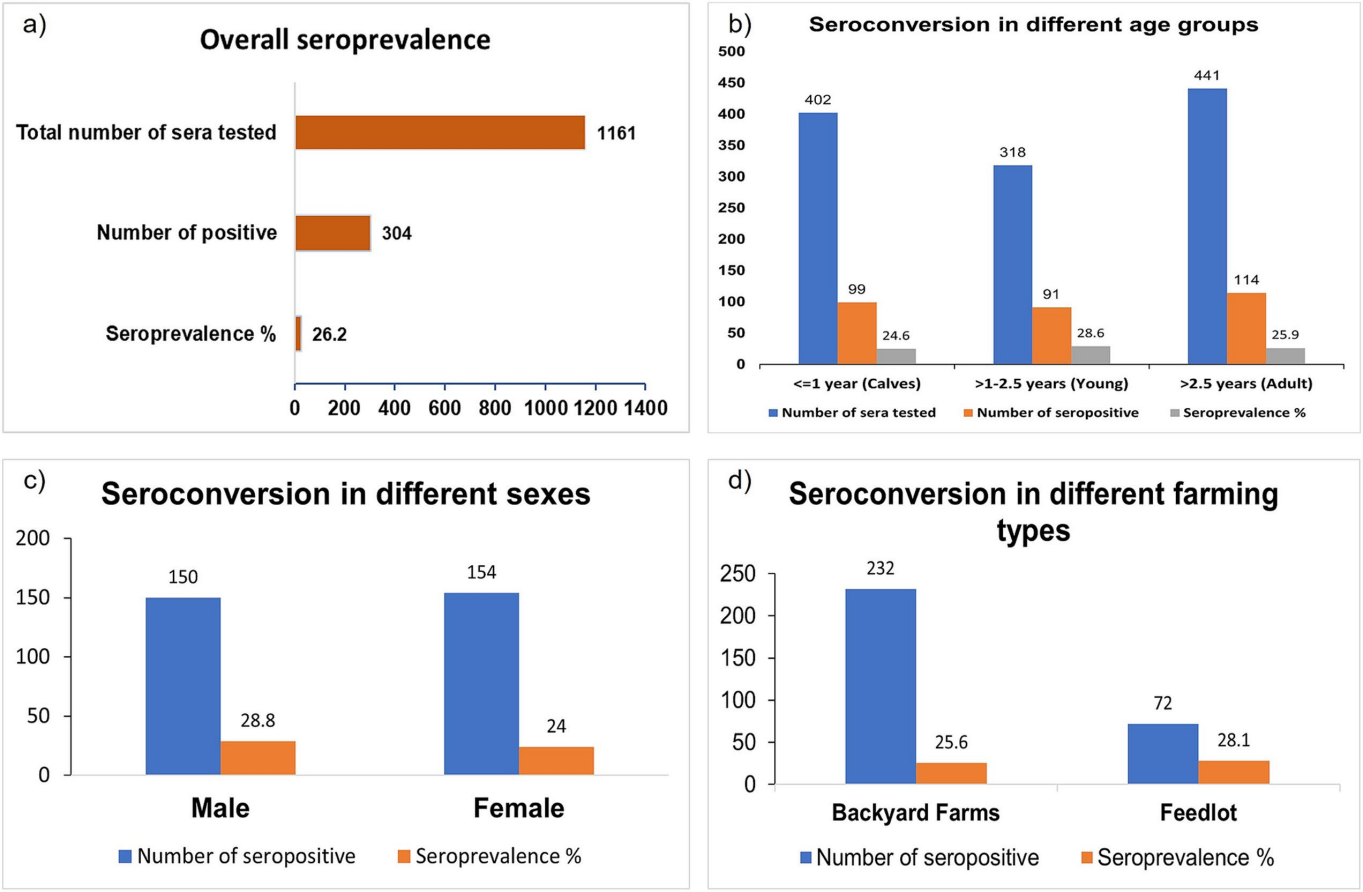
Bar diagram showing the seroprevalence of LSD in the affected area. **(A)** Overall seroprevalence. **(B)** Seroprevalence based on the age of the cattle. **(C)** Seroprevalence based on the sex. **(D)** Seroprevalence based on farming type or size. There is no significant seroconversion differences based on age, sex, and farming type.

The overall seroprevalence of LSD in the study area was 26.20% (Figure 2A). All the studied seroprevalence parameters and their summarized results are listed in Supplementary Table S1. The seroprevalence status in different stages of investigated cattle as well as different states of LSD is presented below.

#### 3.2.1 Seroprevalence according to descriptive epidemiology (age, sex, and farming type)

The following seroprevalences in different age groups were detected: 26.40% in calves, 28.60% in young cattle, and 25.90%, in adults (Figure 2B). Seroprevalence was slightly higher in males (28.80%) than in females (24.00%) animals (Figure 2C) but there was no major difference between males and females. Similarly, the farming type also did not affect as noticed at 25.60% seroprevalence in household cattle and 28.10% in herd cattle (Figure 2D).

#### 3.2.2 Seroprevalence according to clinical disease and vaccination status

The data was further analyzed to investigate the antibody response among LSD-infected, recovered, and clinically healthy in-contact cattle populations. LSD-recovered cattle showed a significantly higher (*p 0.007) seroprevalence rate than clinically healthy and clinically infected populations (Supplementary Table S1). It may take longer for clinically infected cattle to progressively achieve the seroconversion titer after they have fully recovered from the clinical infection, as evidenced by the recovered animal.

**Figure 3:**
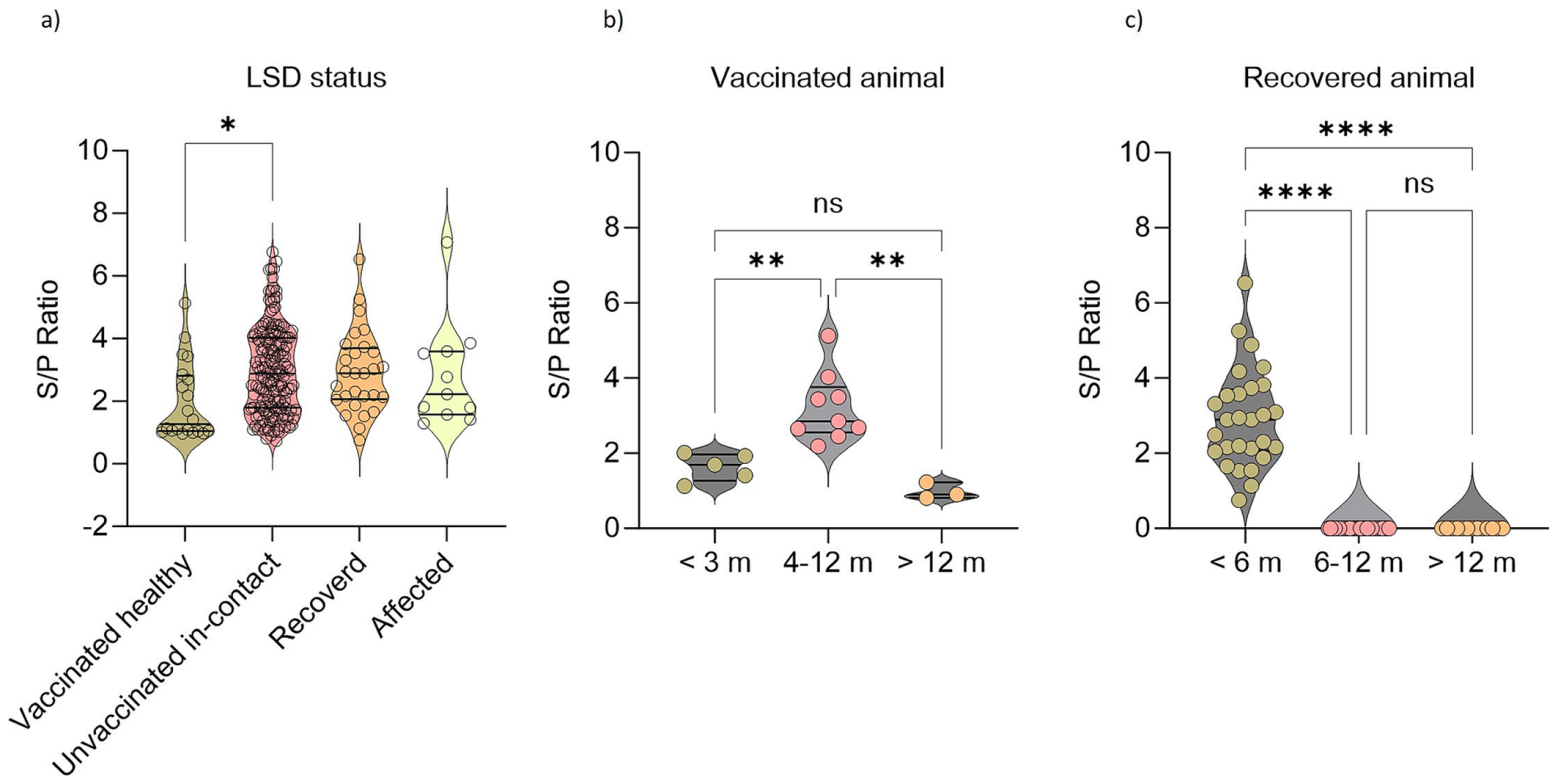
Seroconversion against LSDV in affected, recovered, clinically healthy vaccinated, and unvaccinated cattle. Violin plots showing **(A)** LSD antibody responses in animals based on their disease status. **(B)** LSD antibody responses in vaccinated cattle based on their age groups. **(C)** LSD antibody responses in recovered animals based on their age groups. One-way ANOVA with Bonferroni multiple comparison test. *p ≤ 0.05, **p ≤ 0.01, ****p ≤ 0.0001, ns = not significant.

There was no significant difference between the vaccinated and unvaccinated cattle in antibody response against LSDV. While looking into the antibody titers (S/P ratio: OD sample − ODNC/ODPC − ODNC), clinically healthy unvaccinated in contact with infected cattle showed significantly higher antibody titers than the vaccinated animals (Figure 3A). Among vaccinated cattle significantly higher serological responses were obtained between 4 and 12 months after vaccination (Figure 3B). Interestingly, clinically healthy unvaccinated in-contact cattle showed significant antibody titer as well (Figure 3A). In this study, the antibody response in naturally recovered cattle was limited to within 6 months of infection (Figure 3C). However, this does not mean that the animals would not be protected against the disease.

**Figure 4:**
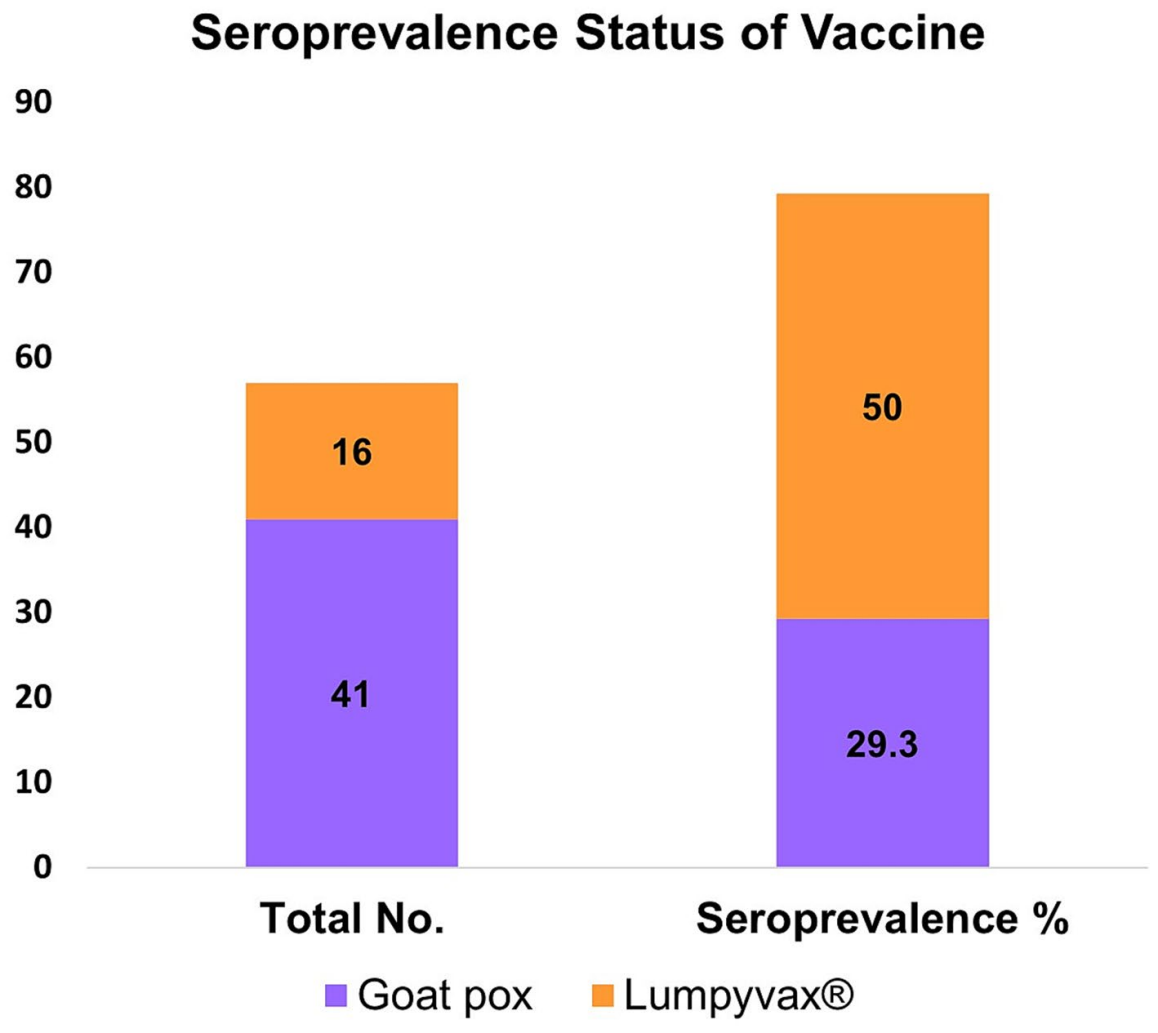
Bar diagram showing the seroconversion against LSDV in vaccinated cattle using two different vaccines Goat pox and Lumpyvax®. Cattle vaccinated with Lumpyvax® showed the highest seroconversion compared to the Goatpox vaccine.

Furthermore, the two different vaccines “Goat Pox” and “Lumpyvax®” used in two different herds were monitored. Serum was tested 10 months after the goat pox vaccination for the herd cattle that had received it. Conversely, serum was taken from the Lumpyvax® receiving herd 9 months after vaccination. Out of the 57 vaccinated animals sampled, 20 seropositive animals were noticed. Seroprevalence of Goat Pox and Lumpyvax® vaccines was 29.3% (12/41) and 50% (8/16) respectively (Figure 4) within the limited sampling of the vaccinated herds.

#### 3.2.3 Seroprevalence in repeated sampling after LSD infection

Among 1,161 samples, 922 samples were collected at D0, 165 samples were at D40, and 75 samples at D70. Two trends in the seroprevalence of animals affected by LSD were observed at three different time intervals (D0, D40, and D70) of blood collection. According to the first trend, the antibody titer is gradually increasing; cattle that have either recovered recently or are still infected have a higher titer (mean = 0.254) at D70 than at D40 and D0. A further trend in cattle that have lived for roughly 10–12 months following vaccination or recuperation is a steady decline in antibody titer (Figure 3).

### 3.3 Detection of LSDV in tissues and body fluids

**Figure 5:**
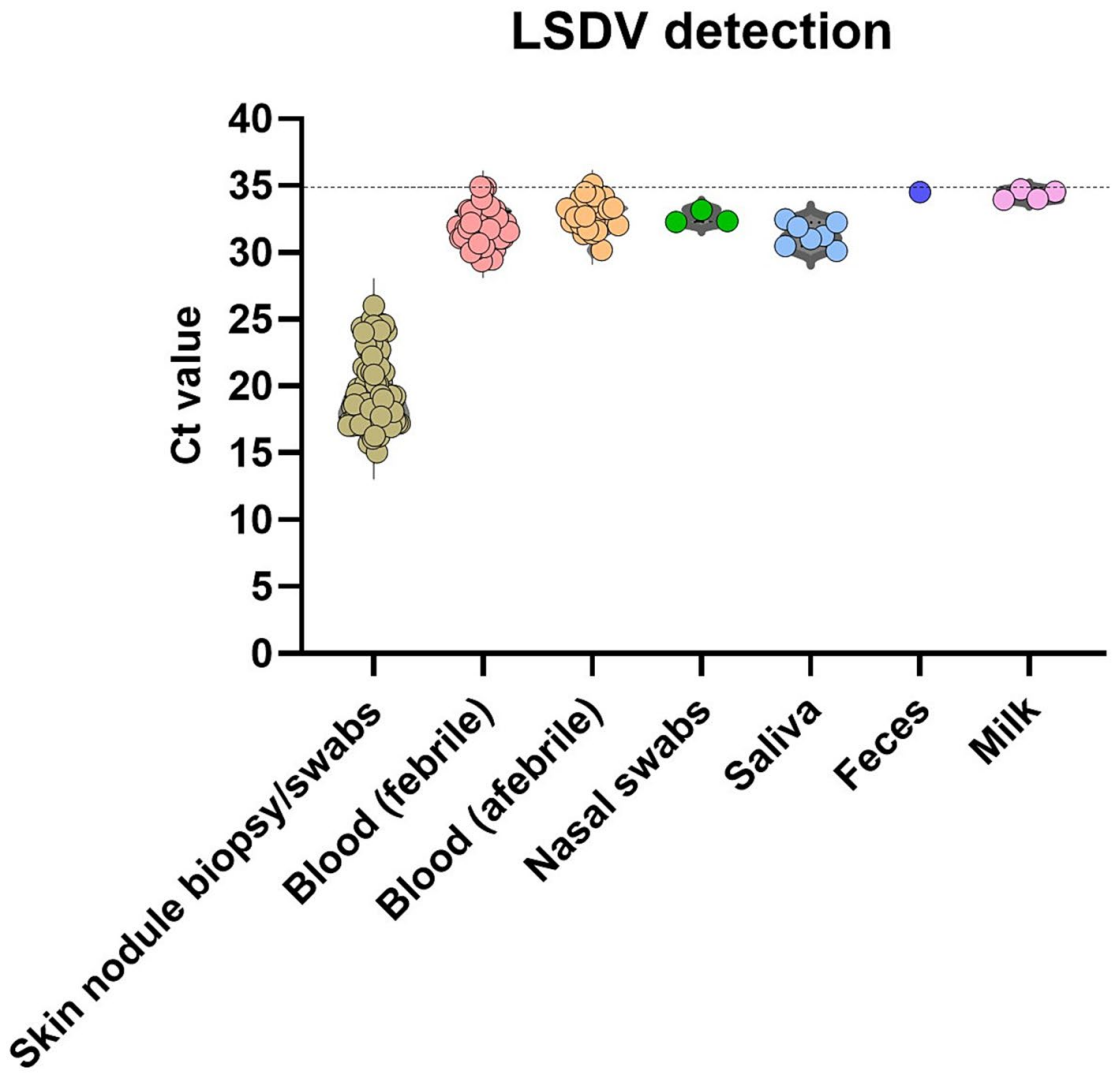
Detection of LSDV genome with qPCR. The scatter plots describe the detection limit (calculated from the Ct values at qPCR) for different positive samples. The level of significance was calculated using a one-way ANOVA test (Tukey’s multiple comparison test). ***p ≤ 0.0001; *p ≤ 0.05.

The LSD viral DNA load was highest in the skin lesions, where all skin biopsies or swabs (*n* = 211) tested positive (100.00%) with Ct values ranging between 14.5 and 26.8. Fever in infected animals usually indicates the presence of viremia. LSDV DNA was more commonly detected in the blood samples taken from animals showing fever (41.6%), than in those with normal body temperature (26.6%). PCR-positive results were less frequently obtained from nasal discharge (15.00%), saliva (17.50%), milk (10.50%), and fecal (6.25%) samples. In addition, the virus concentration in these samples was significantly lower (Ct ranged between 28 and 35) of the virus (Figure 5) than in skin samples.

## 4 Discussion

In this study, LSD seromonitoring was conducted in naturally infected, recovered, and in-contact animals that share the same household with affected cattle, unvaccinated (mostly in the backyard), and vaccinated (feedlot) cattle in study areas using the commercially available ELISA kit. The clinically healthy infected animals can transmit the disease mechanically by vectors or horizontally (35). Therefore, an in-depth cross-sectional study on seroprevalence was developed.

We found that the overall seroprevalence of LSD was 26.20%. It is important to note that, according to multiple previous research studies conducted in the East African regions, the overall seroprevalence of LSD was reported to be 17.40% in Egypt (39), 8.70% in Uganda (4), and 26.50% in Ethiopia (40). Furthermore, compared to reports from Western Wollega, Ethiopia, North-Eastern Ethiopia, and Uganda (4, 41, 42), the seroprevalence of LSD in the current study was significantly higher. This could be brought on by variations in cattle breed, husbandry techniques, seasons, vaccination status, environmental factors, geographic locations, and immune status of cattle populations.

The current study indicated a herd-level seropositivity of 29.30% and an overall backyard-level seropositivity of 20.70%, suggesting that herd cattle exhibited somewhat better immunity. This could be explained by improved herd-level management and biosecurity as well as appropriate vaccination. However, higher individual animal-level seroprevalence has been reported in different agroecological zones in East Africa and Ethiopia (43, 44). It is worth mentioning that no DIVA test was performed to differentiate antibodies produced from vaccination and natural infections.

Considering the age of animals, the seroprevalence was similar between the different age groups and there were no statistically significant variations among the three groups. When comparing such findings agree with the findings of the previous studies (32, 33, 42, 45). Similarly, a significant association between sex and seropositivity to LSD was absent although it could be shown that lactating cows seem to be most susceptible (42).

The seroprevalence of LSDV with disease status (clinically healthy in-contact cattle, infected, and recovered) was a novel parameter examined in the study. The seroprevalence among the three groups differed, where antibody titer with the recovered group showed a higher antibody titer than the clinically healthy and affected group, with statistically significant variations (*p* = *0.007). In addition, the in-contact animals that shared the same household and grassed together with the infected and clinically sick animals demonstrated significant seroconversion but did not show any symptoms. Likely, these animals were subclinically or silently infected and the clinical signs have gone unnoticed. It also indicates that the number of subclinically infected animals can be much greater than previously known. However, the exact mechanism behind the silent infection in that affected area is currently unknown. These animals can spread the disease through insect vectors but on the other hand, they get natural immunity which likely prevents severe disease. This should be considered when animals are tested for import and export.

No previous studies in Bangladesh have considered clinical and vaccination status as a criterion to compare the seroconversion of LSDV. In the present study, seroprevalence of LSDV in the vaccinated cattle population showed seroconversion up to 12 months. Whereas, unvaccinated naturally recovered animals also showed seroconversion for a shorter period (up to 6 months). Development of the short-term humoral immune response against LSDV has been reported earlier (46). Furthermore, sampling from two different herds found that Lumpyvax® has better seropositivity than the goatpox vaccine when the serum was collected post 9 months and 10 months of respective vaccination. The seroconversion in the vaccinated and unvaccinated cattle remained consistent because the study was designed to sample both vaccinated affected herds (herd cattle) and unvaccinated (household cattle) affected and recovered backyard cattle.

Despite immunoresponse shown either by natural infection or post vaccination, both herd and household backyard cattle are getting infection. Clinical and silent infections are ongoing as confirmed by the molecular detection in various samples. This study shows that antibodies are transient and disappear after 6 months of exposure in both sick and recovered animals. This seroconversion might not be as effective as protection against serious infection. On the other hand, the vaccinated herd’s animal level seroprevalence lasted for a maximum of 10–12 months following vaccination. Although we could find any baseline information of such observation, this may due to the management and restrict movement of the herd level cattle than the household backyard cattle rearing system. Additionally, vaccination coverage is very limited in Bangladesh thus, most of the cattle population remains unvaccinated thus the observation did not represent the whole cattle population in Bangladesh. Moreover, a short-term seroconversion of the recovered or infected cattle implies that the virus may persist in the local cattle population and that LSDV incidence is repeated. Therefore, it is also important to focus seromonitoring on the vast cattle populations and wildlife reservoirs. It is strongly advised to use mass coverage, either with homologous (lumpy skin disease vaccine), or heterogeneous (goat pox vaccine) vaccination strategies.

## 5 Conclusion

We provide an in-depth view of LSDV seroprevalence in Bangladesh. Our study provides important baseline data on the incidence of LSD and the post-infection antibody response. The disease is spreading into new areas and is found to have a moderate seroprevalence. Recurring infections are occurring in the same area. It is necessary to take consideration of mass vaccination with the proper vaccines, applied to all cattle at the household and herd levels. Effective control strategies require more research to evaluate the disease status and antibody response across cattle populations.

## Data Availability

The original contributions presented in the study are included in the article/Supplementary material, further inquiries can be directed to the corresponding authors.

## Supplementary material

SUPPLEMENTARY FIGURE S1 The geographical location of the sampling area from the household and herd cattle taken into consideration for this study. The yellow color designates the sample collection area, which is displayed in the right panel as a closer view of the areas.
