# Supplementary figures and images for: Serological response to lumpy skin disease in recovered and clinically healthy vaccinated and unvaccinated cattle of Bangladesh
Source: Front Vet Sci. 2025 Feb 17;12:1535600. doi: 10.3389/fvets.2025.1535600 (PMC11873106; doi:10.3389/fvets.2025.1535600)

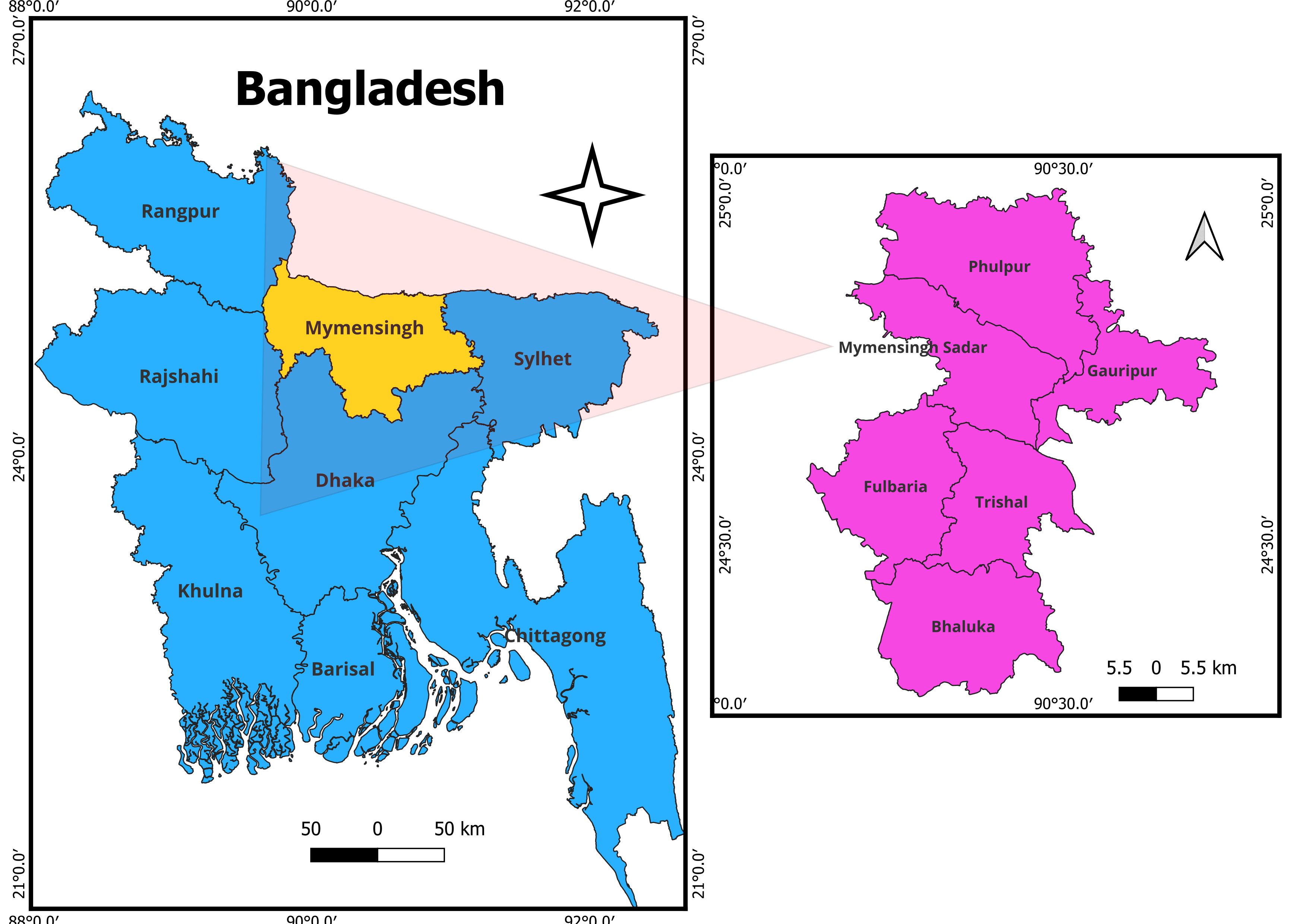

Supplement: SUPPLEMENTARY FIGURE S1 — The geographical location of the sampling area from the household and herd cattle taken into consideration for this study. The yellow color designates the sample collection area, which is displayed in the right panel as a closer view of the areas. [file Image_1.TIF]
