# Supplementary material for: Serological response to lumpy skin disease in recovered and clinically healthy vaccinated and unvaccinated cattle of Bangladesh
Source: Front Vet Sci. 2025 Feb 17;12:1535600. doi: 10.3389/fvets.2025.1535600 (PMC11873106; doi:10.3389/fvets.2025.1535600)
Supplement: Supplementary file 2 [file Table_1.DOCX]

Supplemental Table S1: Seroprevalence of lumpy skin disease (LSD) in different studied parameters.

| **Parameters** | **Number of sera tested** | **Number of seropositive** | **Seroprevalence (%)** | **P value** |
| --- | --- | --- | --- | --- |
| **Age** | | | | |
| <=1 year (Calves) | 402 | 99 | 24.60 | 0.596 |
| >1-2.5 years (Young) | 318 | 91 | 28.60 |  |
| >2.5 years (Adult) | 441 | 114 | 25.90 |  |
| **Sex** | | | | |
| Male | 520 | 150 | 28.80 | 0.107 |
| Female | 641 | 154 | 24.00 |  |
| **Farming Type** | | | | |
| Household | 905 | 232 | 25.60 | 0.328 |
| Herd | 256 | 72 | 28.10 |  |
| **Disease Status** | | | | |
| Clinically affected | 211 | 38 | 18.00 | *0.007 |
| Recovered | 267 | 107 | 40.07 |  |
| Clinically healthy | 683 | 159 | 23.27 |  |
| **Vaccination Status** | | | | |
| Unvaccinated | 1104 | 289 | 26.20 | 0.078 |
| Vaccinated | 57 | 15 | 26.80 |  |
| **Total** | **1161** | **304** | **26.20** |  |
